# Pooled estimate of vitamin D deficiency among pregnant women in India: a systematic review and meta-analysis

**DOI:** 10.1186/s41043-021-00253-y

**Published:** 2021-06-29

**Authors:** Angeline Jeyakumar, Vidhya Shinde, Reshma Ravindran

**Affiliations:** 1 Interdisciplinary School of Health Sciences, Savitribai Phule Pune University, Maharashtra, India; 2 School of Hospitality Management, University of Johannesburg, Johannesburg, South Africa

**Keywords:** Vitamin D deficiency, Pregnant women, Systematic review, Meta-analysis

## Abstract

**Background:**

Vitamin D deficiency among pregnant women is a public health concern globally. In India, individual studies report high prevalence. However, lack of national data masks the true burden. This work determined the pooled prevalence of vitamin D deficiency among pregnant women in India through a systematic review of literature and meta-analysis.

**Methods:**

Three different search engines yielded 15 eligible articles. Study quality was assessed by 10 different criteria and summary of study quality was categorized as per Cochrane standards. Meta-analysis was performed to estimate pooled prevalence of vitamin D deficiency among healthy pregnant women and heterogeneity among selected studies. A sample of *n* = 4088 was used to study the pooled prevalence among pregnant women.

**Results:**

The random effects combined estimate was 32.35% (95% CI, (12.58–117.48). High heterogeneity (tau^2^ = 0.39, I^2^ = 100%) and high risk of bias was observed among the selected studies. The test for overall effect was observed to be z = 2.54(*P* = 0.01).

**Conclusion:**

Pooled estimate > 30% emphasizes the need for screening through antenatal care services and initiate preventive measures to address the deficiency.

## Introduction

Vitamin D has emerged as a micronutrient of concern due to widespread prevalence of deficiency [1]. Among the different definitions, Endocrine Society defines deficiency of vitamin D as serum levels of 25-hydroxyvitamin D (25[OH]D) below 20 ng/ml and levels between 20 and 30 ng/ml as insufficient [2]. The global prevalence of deficiency or insufficiency ranges between 54–100% and 39–76%, respectively [3]. Mild to severe deficiencies have been reported both in developed as well as third world countries [4]. Among European countries, Belgium reports > 70% prevalence, while tropical countries in Asia with abundant sunshine report even higher prevalence (> 80%) [4–6]. Compared to Asia (80%), African countries show less prevalence (30%). Among Asian countries, in India, the prevalence of vitamin D deficiency among healthy pregnant women is reportedly high [4, 7]. Individual studies report 93% prevalence in Delhi, 97% in Bangalore, Karnataka, and 94% in Mumbai, Maharashtra [6, 8, 9]. High prevalence has been reported among women in reproductive age group both in rural and urban areas, as well as across economic classes [4].

The physiological role of vitamin D implicated beyond bone health evoked extensive research with this vitamin. From a maternal and child health perspective, its role in fertility and conception, pathogenesis in preterm birth, gene transcription in placenta, and immune function are widely researched [10–14]. Deficiency in pregnancy is known to increase risk of pre-eclampsia, gestational diabetes mellitus, preterm birth, and other tissue-specific conditions [1, 11]. Moreover, vitamin D status of neonates and infants is affected by vitamin D levels of mothers [15, 16]. Lactation further increases requirements and severe deficiency has been reported during this phase too [17–20]. As per the guidelines of Endocrine Society, poor vitamin D status in adolescence and increased requirements during pregnancy make the reproductive phase vulnerable [2, 21]. Unlike other vitamins that are obtained through foods, most of the foods commonly consumed are poor sources of vitamin D. The World Health Organization has emphasized the importance of investigating this vitamin as it affects pregnancy outcome [1]. The paucity of national data and high prevalence as per regional evidence identifies the need to estimate the burden among pregnant women in India. The present work is a systematic review and meta-analysis to determine the combined estimate of vitamin D deficiency among healthy pregnant women in India.

## Methods

Standard protocols for systematic review writing by Khan and coworkers [22] and Preferred Reporting Items for Systematic Reviews and Meta-Analyses (PRISMA) guidelines [23] were followed.

### Preliminary research and idea validation

To ensure validity of the chosen topic and to avoid duplication of work, we performed a preliminary search in PubMed with search terms viz. vitamin D deficiency/insufficiency + pregnant women + India. As we did not come across systematic review and meta-analysis for vitamin D deficiency among pregnant women or national prevalence data in India, we chose to perform this systematic review and meta-analysis. We also found substantial responses to these search terms that enabled us to progress with this research.

### Literature search

**Fig. 1:**
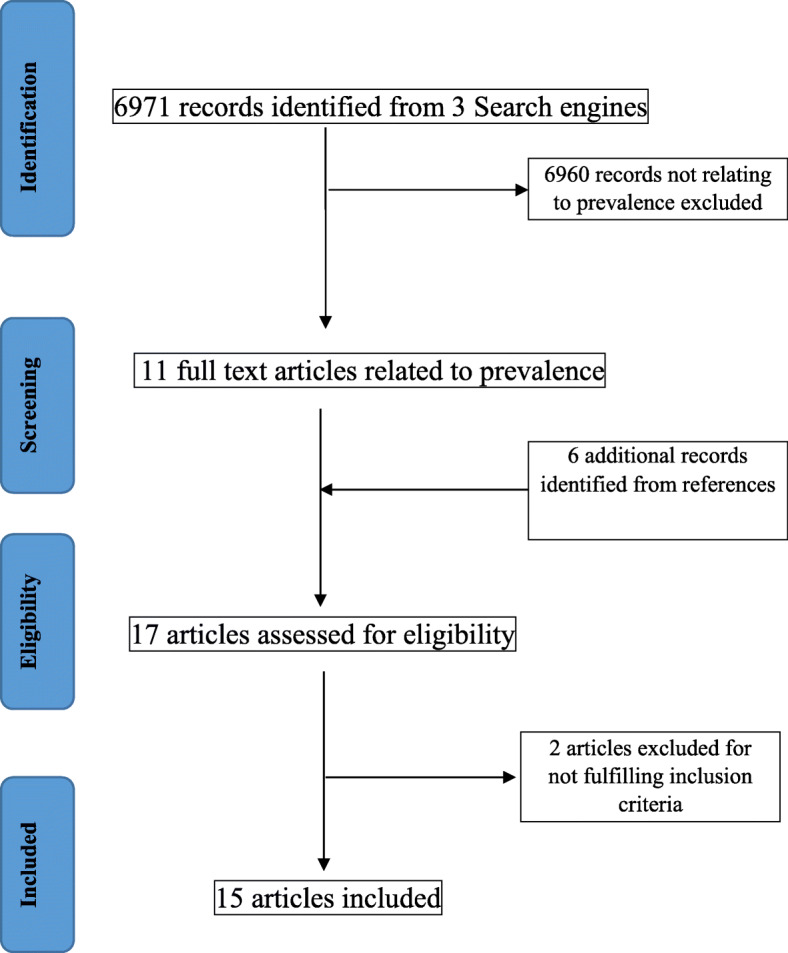
PRISMA chart: Search strategy and selection of studies

A systematic literature search was performed by two researchers independently in electronic databases that included PubMed, Google Scholar, and Web of Science in November 2018. The search terms used were ("epidemiology"[Subheading] OR "epidemiology"[All Fields] OR "prevalence"[All Fields] OR "prevalence"[MeSH Terms]) AND ("vitamin D deficiency"[MeSH Terms] OR "25 OH Vitamin D levels"[All Fields]) AND ("pregnant women"[MeSH Terms] OR ("pregnant"[All Fields] AND "women"[All Fields]) OR "pregnant women"[All Fields]) AND ("india"[MeSH Terms] OR "india"[All Fields])) AND ("2007/12/03"[PDat] : "2018/11/29"[PDat]) (Fig. 1).

### Study selection

Applying selection criteria (a) studies that were original articles, (b) published in English language, (c) study designs that were observational, intervention studies that provided baseline information on vitamin D levels of healthy pregnant women, (d) India as study location, (e) studies that determined the prevalence of vitamin D deficiency among pregnant women across gestational age, irrespective of parity were selected. (f) Time frame for literature selection was restricted to those published between 2005 and 2018. (g) As the objective of the present review is to study vitamin D deficiency among pregnant women, studies that recruited pregnant women from hospitals were included. Reference lists of the selected articles were used for manually identifying relevant literature. Full-text articles that were unavailable and data required for participants in specific age groups were obtained from authors on request. All papers were screened and verified by two researchers independently.

### Exclusion criteria

(i) Earlier work had used > 30 sample as a selection criterion [24]. In our search, the least sample in the eligible studies was *n* = 20 and the next higher sample was *n* = 50. As smaller studies increase the risk of bias, studies with sample size less than 50 were excluded.

(i) Studies that reported vitamin D deficiency associated with specific disease conditions and (ii) eligible studies from which data if unavailable from authors after request were excluded.

### Data extraction

Full texts of the selected articles were retrieved. To avoid publication bias, only peer-reviewed published studies were included. The outcome of interest was combined estimate of vitamin D deficiency among pregnant women in India. For this, the estimated prevalence of deficiency was recorded from every selected study. In addition, associated variables that describe the study characteristics such as study design, study setting, socio-demographic and economic status, and criteria used to categorize deficiency and sufficiency, season of study, maternal characteristics of pregnant women such as gestational age, and parity were recorded. Data was extracted and entered in Microsoft Excel in duplicate by RR and VS. Disagreement in selection of articles and data clarification if any was verified by third reviewer AJ.

### Assessment of study quality

Criteria proposed by Hoy and coworkers [25] for prevalence studies were applied to assess the risk of bias in the selected articles. This model applies 10 criteria for assessment of risk of bias. Applying this, the papers were assessed for representation of population, sampling, random selection, non-response bias, data collected directly from subjects, case definition, reliability and validity of the method used, mode of data collection whether similar, length of shortest prevalence period, and numerator and denominator. The summary of study quality was categorized as per Cochrane standards [26] as low (all 10 criteria assessed to have low risk), moderate (at least two criteria showing high risk), and high risk (more than two criteria showing high risk).

### Statistical analysis

Review manager [27] software version 5.3 was used to obtain a forest plot to demonstrate the degree of heterogeneity among the selected articles. The software uses Chi^2^_,_ I^2^_,_ and Tau^2^ to study heterogeneity. Estimating pooled prevalence is a testing strategy where prevalence from a number of studies are aggregated into a single sample (or pool), which is then evaluated for the prevalence of interest [28]. In this review, reported prevalence in individual papers was extracted, log transformed, and standard error of proportion of prevalence was estimated. Considering the variation in the selected prevalence studies, and not assuming a uniform effect size in the selected studies, random effects model was used to perform meta-analysis. This model prevents one or few studies influencing the overall estimate and allows more balance in the relative weights of the studies [29]. The P value is the probability from chi-square statistic calculated using estimates of individual study weight, effect size, and overall effect size [30]. Publication bias was assessed using a funnel plot. Asymmetry in the distribution of studies in the funnel plot indicates the extent of bias.

## Results

Literature search using specific search terms on prevalence of vitamin D deficiency among pregnant women in India identified 6971 articles. After screening titles and abstracts for relevance and excluding duplicates, 6960 articles were excluded as they did not match the selection criteria. This yielded 11 relevant articles. Six additional records were obtained from references cited within these articles. A total of 17 articles with sample size ranging from 50 to 568 were assessed for eligibility as per the selection criteria that further resulted in exclusion of two articles. Thus, 15 primary studies among pregnant women were included for the review.

**Fig. 2:**
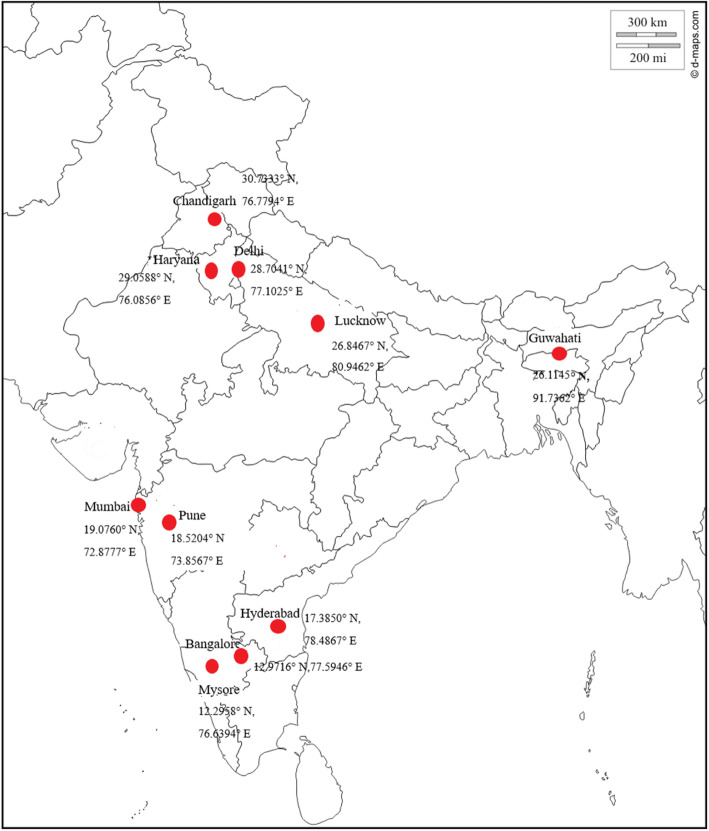
Map showing studies selected from Indian states and their coordinates

Figure 2 shows the states where the studies were carried out and their coordinates on the Indian map. Nine out of 15 studies were conducted in Northern India. Five studies were conducted in south and one study each in west and north-east India. Table 1 describes the articles selected for the review. In all, the sample size in the studies ranged from 50 to 568. Of the 15 selected studies, 6 were cross sectional and the other 8 were prospective cohorts, and a randomized control trial. All studies were hospital based excluding Sahu’s [33] work which was population based with calculated sample size. Table 2 describes the maternal characteristics of selected studies. The age of pregnant women ranged from 18 to 40 years in all the papers reviewed. Socioeconomic status of the study group ranged from upper, middle, and lower income groups. Six out of 15 studies provided information on the educational status of women. Work done by Ajmani and coworkers [35] described the distribution of women as per their level of education. Educational status of participants from other studies ranged from illiterate, primary education to graduates. Study setting was rural, urban, or combined representation of both rural and urban settings.

**Table 1:** Description of studies among pregnant women in India

References	Location	Sample size	Study design Prevalence
Sachan et al. 2005 [31]	Lucknow (North )	207	Cross-sectional 66.6%
Farrant et al. 2009 [32]	Mysore (South )	559	Cross-sectional 66.0%
Sahu et al. 2009 [33]	Lucknow (North)	139	Cross-sectional 74.0%
Marwaha et al. 2011 [7]	Delhi (North)	541	Cross-sectional 96.30%
Jani et al. 2014 [9]	Mumbai (West )	150	Cross-sectional 94.0%
Singla et al. 2015 [34]	Chandigarh (North)	304	Prospective cohort 92.11%
Ajmani et al. 2016 [35]	Delhi (North)	200	Cross-sectional 37.50%
Sharma et al. 2016 [36]	Delhi (North)	418	Prospective cohort 34.54%
Krishnaveni et al. 2011 [37]	Mysore (South)	568	Prospective cohort 66.0%
Veena et al. 20162017 [38]	Mysore (South)	468	Prospective cohort 66.8%
Nandal et al. 2016 [39]	Haryana (North)	60	Prospective cohort 93.75%
Kumar et al. 2015 [40]	Bengaluru (South)	106	Prospective cohort 70.70%
Chary et al.2015 2014 [41]	Hyderabad (South)	153	Prospective cohort 52.2%
Dasgupta et al. 2012 [42]	Guwahati (North-East)	50	Cohort 42.00%
Sablok et al. 2015 [43]	Delhi (North)	165	RCT 77.50%

*ELISA* enzyme-linked immunosorbent assay, *CI* confidence interval, *RCT* randomized control trials

**Table 2:** Description of associated variables in studies selected for estimating prevalence among pregnant women (*n* = 15)

Study	Age group/mean age	Socio-economic status	Education	Rural/urban	Parity	Trimester	Exposure to sunlight	Seasons of study
Ajmani et al. 2016 [35]	20–25	Lower, upper lower, lower middle, upper middle	Illiterate, primary level, graduate	Urban	Multi-gravida and Primigravida	All trimesters	NM	NM
Farrant et al. 2009] [32]	20–26	Upper, lower	NM	Urban	NM	< 32 weeks of pregnancy	NM	Summer, Winter
Jani et al. 2014 [9]	26.7 ± 4.1	NM	NM	Rural	NM	2nd trimester	Summer: 35.4 ± 15.9 h/day * %BSA	Summer, Winter
Marwaha et al. 2011 [7]	19–30	Lower middle	NM	Urban	1, 2, and > 2	All trimesters	1st trimester: 10–60 min 2nd trimester: 10–60 min 3rd trimester; 10–20 min	Summer, Winter
Sachan et al. 2005 [31]	24 ± − 4.1	Lower, middle	NM	Rural and urban	NM	3rd trimester	Urban: 4.1 ± 3.2 h/day*%BSA Rural: 9.7 ± 8.1 h/day*%BSA	Autumn
Sahu et al. 2009 [33]	20–25	Lower, upper	NM	Rural and urban	< 3	3rd trimester	Mean 14:00 ± 2 h	Spring summer
Sharma et al. 2016 [36]	22–23	Lower, upper lower, lower middle upper middle	Both educated and not educated	Urban	Primigravida	Full term	NM	Summer, Winter
Singla et al. 2015 [34]	18–35	Upper, upper middle, lower, lower middle	NM	Urban	Nulliparous, 1 and 2	2nd trimester	Summer: shorter ≤ 30 min Longer > 30 min Winter: Shorter ≤ 90 min Longer > 90 min	Summer, Winter
Krishnaveni et al. 2011 [37]	24 ± 4.3	Lower	NM	NM	NM	3rd trimester	NM	NM
Veena et al. 2017 [38]	23.9 ± 4.3	Lower	< 10(34.9% − 10(31.7%) > 10(33.4%	NM	1, 2, and > 2	3rd trimester	NM	NM
Nandal et al. 2016 [23, 39]	30.83 ± 4.0	upper	NM	urban	NM	2nd trimester	NM	NM
Kumar et al. 2015 [40]	NM	NM	NM	NM	NM	NM	NM	NM
Chary et al. 2015 [41]	24.5 ± 2.6	Upper, upper middle, lower, lower middle	Illiterate, primary level, High School Post high school	Rural and urban	NM	3rd trimester	< 60 min	NM
Dasgupta et al. 2012 [42]	20–40	NM	NM	NM	NM	1st trimester	33 ± 9.07%	Summer, rainy
Sablok et al. 2015 [43]	NM	Lower, middle	NM	NM	Primigravida	2nd trimester	< 1 h/day > 4 h/day	NM

*NM* not mentioned

Parity was described in 7 out of 15 articles [31–44] accordingly pregnant women were primi or multi-gravida, nulliparous, or had parity less than three. Women across trimesters were recruited in two articles [35, 44]. Women in second trimester were enrolled in five studies [9, 32, 34, 39, 43] and four studies were conducted during the last trimester of pregnancy [33, 37–40, 42, 43]. Data pertaining to sunlight exposure was provided by 8 out of 15 papers. Of these, two papers [9, 42] provided a direct estimation of sunlight exposure by duration to percent body surface area and while two [9, 34] provided duration exposed specifically in summer and winter. Ajmani and coworkers [35] worked among burka-clad pregnant women and provided information about sunlight exposure indirectly by the number of hours of outdoor activity and use of sun screens and skin complexion. Eight studies mentioned the seasons of study [9, 32, 34, 36, 42, 44]; however, seven studies did not mention the season of study.

Table 3 describes the techniques used in determining the vitamin D levels and the estimated prevalence in the selected studies. Serum was used as the sample for vitamin D estimation in all the studies. Among the techniques used, ELISA [31, 32, 34, 35, 39, 44] and radioimmunoassay were used in six studies [32–34, 36–40, 42, 43] and chemiluminescent assay and HPLC [41] and LC-MS/MS [40] by one study each. Except Farrant’s work [32] (2009), all studies were limited in detail pertaining to standardization and validation of methods. Majority of the studies (13 out of 15)) used 20 ng/ml as the cuff for defining deficiency, although some studies used [35, 39] 10 or 12 ng/ml defining severe deficiency. Table 4 summarizes risk of bias (RoB) of the selected papers among pregnant women. Sahu’s work [33] was the only population-based study that estimated prevalence based on sample size calculation. Therefore, his work scored low risk in domains pertaining to (i) population representation and (ii) numerator and denominator. All other articles scored high risk in the above-mentioned domains. In all, 13 out of 15 selected studies were categorized as high risk as at least two domains were categorized as high risk, one study each were categorized as moderate and low risk, respectively.

**Table 3:** Mean levels of serum 25 (OH) D among pregnant women

Study	Vitamin D estimation method	Mean serum 25(OH) D	25 (OH) D ranges in serum		
			Deficiency	Insufficiency	Sufficiency/adequacy
Ajmani et al. 2016 [35]	ELISA	23.25 ng/ml ± 18.49	< 20 ng/ml	20–30 ng/ml	> 30 ng/ml
Farrant et al. 2009 [32]	Radioimmunoassay	Median: 15.12 ng/ml	< 20 ng/ml	NM	NM
Jani et al. 2014 [9]	Chemiluminescent immunoassay	10.6 ng/mL	< 20 ng/ml	<20–30 ng/ml	30 ng/ml
Marwaha et al. 2011 [7]	ELISA	9.28 ng/ml	< 20 ng/ml	NM	NM
Sachan et al. 2005 [31]	Radioimmunoassay	14.93 ng/mL	< 20 ng/ml	NM	Normal: 20–80 ng/ml nmol/L
Sahu et al. 2009 [33]	Radioimmunoassay	15.12 ng/ml ± 7.92	< 20 ng/ml	NM	30 ng/ml
Sharma et al. 2016 [36]	ELISA	Deficiency: 7.10 ± 1.49 ng/ml	Severe < 10 ng/ml	Deficient < 20 ng/ml)	Normal 32–100 ng/ml
Singla et al. 2015 [34]	ELISA	Median: 7.9 ng/ml (IQR 5.7, 12	< 20 ng/ml	< 20–30 ng/ml	30 ng/ml
Krishnaveni et al. 2011 [37]	Radioimmunoassay	15.6 ng/ml	< 20 ng/ml	NM	NM
Veena et al. 2017 [38]	Radioimmunoassay	NM	< 20 ng/ml	NM	NM
Nandal et al. 2016 [39]	ELISA	11.98 ng/ml	< 12 ng/ml	12–20 ng/ml	20–30 ng/ml
Kumar et al. 2015 [40]	LC-MS/MS	16.3 ng/ml	< 20 ng/mL	NM	NM
Chary et al. 2015 [41]	HPLC	NM	< 19 ng/mL	20–29 ng/mL	> 30 ng/mL
Dasgupta et al. 2012 [42]	Radioimmunoassay	38.4 ± 18.37 ng/ml	< 20 ng/ml	NM	NM
Sablok et al. 2015 [43]	ELISA	18.44 ng/ml	< 20 ng/ml	< 20–30 ng/ml	30 ng/ml

*NM* not mentioned, *IQR* inter quartile range, *SD* standard deviation

**Table 4:** Risk of bias and summary of risk of selected studies

Risk of bias	Krishnaveni et al. 2011 [37]	Veena et al. 2017 [38]	Nandal et al. 2016 [39]	Dasgupta et al. 2012 [42]	Sachan et al. 2005 [31]	Sahu et al. 2009 [33]	Farrant et al. 2009 [32]	Sharma et al. 2016 [36]	Marwaha et al. 2011 [7]	Jani et al. 2014 [9]	Ajmani et al. 2016 [35]	Chary et al. 2015 [41]	Kumar et al. 2015 [40]	Singla et al. 2015 [34]	Sablok et al. 2015 [43]
Representation of data	High risk	High risk	High risk	High risk	High risk	Low risk	High risk	High risk	High risk	High risk	High risk	High risk	High risk	High risk	High risk
Sampling	High risk	High risk	High risk	High risk	High risk	low risk	High risk	high risk	High risk	High risk	High risk	High risk	High risk	High risk	High risk
Random selection	Low risk	Low risk	Low risk	Low risk	Low risk	Low risk	Low risk	Low risk	Low risk	Low risk	Low risk	Low risk	Low risk	Low risk	Low risk
Non-response bias	High risk	High risk	Low risk	Low risk	High risk	Low risk	High risk	High risk	Low risk	Low risk	Low risk	Low risk	High risk	High risk	Low risk
Data collected directly from subject	Low risk	Low risk	Low risk	High risk	Low risk	Low risk	Low risk	Low risk	Low risk	Low risk	Low risk	Low risk	Low risk	Low risk	Low risk
Case definition	Low risk	Low risk	Low risk	Low risk	Low risk	Low risk	Low risk	Low risk	Low risk	Low risk	Low risk	Low risk	Low risk	Low risk	Low risk
Reliability and validity of method	Low risk	Low risk	Low risk	Low risk	Low risk	Low risk	Low risk	Low risk	Low risk	Low risk	Low risk	Low risk	Low risk	Low risk	Low risk
Same mode of data collected	Low risk	Low risk	Low risk	Low risk	Low risk	Low risk	Low risk	Low risk	Low risk	Low risk	Low risk	Low risk	Low risk	Low risk	Low risk
Length of the shortest period of the prevalence	Low risk	Low risk	Low risk	Low risk	Low risk	Low risk	Low risk	Low risk	Low risk	Low risk	Low risk	Low risk	Low risk	Low risk	Low risk
Numerator and denominator	High risk	High risk	High risk	High risk	High risk	Low risk	High risk	High risk	High risk	High risk	High risk	High risk	High risk	High risk	Low risk
Summary*	High risk of bias	High risk of bias	High risk of bias	High risk of bias	High risk of bias	Low risk of bias	High risk of bias	High risk of bias	High risk of bias	High risk of bias	High risk of bias	High risk of bias	High risk of bias	High risk of bias	Moderate risk of bias

Low risk of bias: all criteria met (i.e., low for each domain). *Moderate risk of bias: one to two criteria not met (i.e., high for each domain), High risk of bias: more than two criteria not met (i.e., high for each domain)

**Fig. 3:**
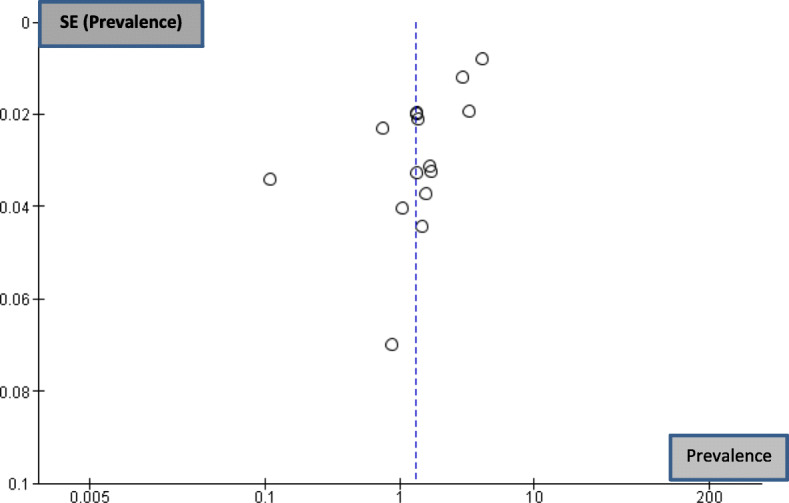
Funnel plot of individual studies selected for meta-analysis

**Fig. 4:**
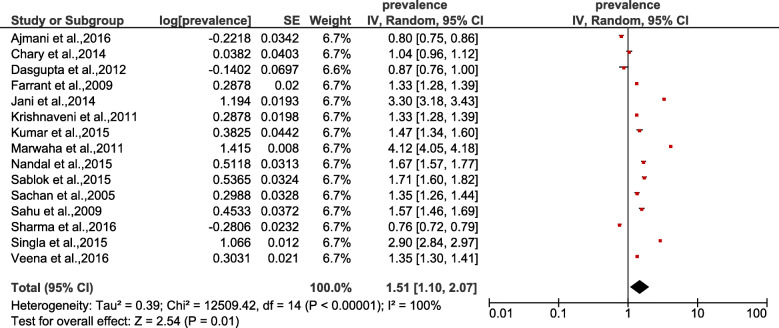
Forest plot showing pooled estimate of vitamin D deficiency among pregnant women

The asymmetrical distribution of studies in the forest plot (Fig. 3) provides a visual representation of publication bias. Figure 4 shows the forest plot derived for the selected studies. The prevalence of vitamin D deficiency among pregnant women ranged from 34.45 to 96.30%. High heterogeneity was observed among the studies (Tau^2^ = 0.39, chi^2^ = 12509.42, df = 14, *p* = < 0.00001, I^2^ = 100%). The test for overall effect was observed to be Z = 2.54(*p* = 0.01). As per categorization of heterogeneity by Higgins et al. 2003 [45], I^2^ > 75% indicates considerable heterogeneity. This indicates large variation among included studies. The random effects combined estimate for overall prevalence was 32.35%, 95% CI, 12.58–117.48).

## Discussion

Vitamin D deficiency among women in reproductive age has gained public health attention in recent years. The estimated pooled prevalence as per this review was 32.35% among healthy pregnant women. As per current literature evidence, a population prevalence > 20% is considered a public health problem that calls for immediate intervention  [46]. Data from individual studies in developing countries report high prevalence in Bangladesh (81%), Lahore, in Pakistan (73%), Beijing (40%), and Malaysia (90%)  [46–49]. Another systematic review among Indian pregnant women by Tasset [50] reported 66–98% prevalence; however it was not a pooled estimate. These findings underscore the unmet requirements that increase vulnerability during the reproductive phase.

Besides high requirements in pregnancy, geographical location and climate affect vitamin D status. While lack of sunshine contributes to low vitamin D status in developed countries, poor living conditions, economic status, and cultural factors affect those in developing countries despite adequate sunshine. For instance, in Europe and Japan, low and high prevalence were reported in summer and winter, respectively [51, 52]. Whereas in developing countries, urbanization and transition increase risk for poor vitamin D status irrespective of season [53, 54] Although women in lower socioeconomic strata are highly susceptible, women from higher socioeconomic status who preferred indoors too were at equal risk [55]. Cultural practices such as women covering maximum body surface and veiling prevents maximum sun exposure [56]. Dark skin among south Asians further limits absorption of vitamin D. In resource poor settings houses are closely packed with no direct sunlight within their dwellings and high level of air pollution aggravates vulnerability [57]. High prevalence of deficiency has been reported among migrant women in developing countries [58]. The above factors associated with poor vitamin D status are commonly observed in developing countries as a consequence of urbanization [1, 59–64]. However, rural areas as place of residence did not decrease the risk of vitamin D deficiency. Poor access to nutrient dense foods increased risk in these settings as well [65]. Among maternal characteristics, multi-parity combined with low vitamin D intake is known to increase risk of deficiency [16, 66, 67]

A global summary of maternal and newborn vitamin D status reports 87% deficiency among pregnant women in Southeast Asia  [68], while pooled estimates show lower prevalence. Varied estimates of prevalence arise due to variations in techniques and difference in defining deficiencies and geographical variations [69, 70].

Dearth of national level data in developing countries masks the true burden of this deficiency and limits comparison. National surveys have not focused on screening vitamin D levels of pregnant women for deficiency. In India, the national guidelines recommend 500 mg elemental calcium and 250 IU vitamin D3 twice a day to meet the increased requirements in pregnancy [71]. However, considering the low quality of available evidence between deficiency state and critical pregnancy outcome there exist no recommendation for vitamin D supplementation as part of routine antenatal care [72–74]. This meta-analysis has provided a pooled estimate in the absence of a national prevalence of vitamin D deficiency. However, it suffers from the following limitations: despite finding eligible studies some studies were excluded due to non-response from authors. Therefore, it is likely that the studies selected for this review are not a representation of the available literature. Although funnel plot was created using RevMan software, statistical test for publication bias could not be performed using this software. Sensitivity analysis could not be performed as prevalence from the excluded papers could not be derived. The high risk of bias due to low power of the selected studies and the time period applied for selecting studies further added to the study limitation.

## Conclusion

The pooled estimate of vitamin D deficiency according to the selected Indian literature identifies a significant percentage of deficiency among pregnant women. Screening of women in reproductive age would identify the magnitude of deficiency to promote early intervention. Vitamin D deficiency is a potentially preventable micronutrient deficiency and high prevalence calls for public health strategies to address this serious issue.

## Data Availability

Please contact author for data requests.

## References

[1] World Health Organization. Guideline: vitamin D supplementation in pregnant women.2012; Available in http://www.who.int/nutrition/publications/micronutrients/guidelines/vit_d_supp_pregnant_women/en/ Accessed December 2018.

[2] Holick MF, Binkley NC, Bischoff-Ferrari HA, Gordon CM, Hanley DA, Heaney RP, Weaver CM (2011). Evaluation, treatment, and prevention of vitamin D deficiency: an Endocrine Society clinical practice guideline. J Clin Endocrinol Metab.

[3] Van der Pligt P, Willcox J, Szymlek-Gay E, Murray E, Worsley A, Daly R (2018). Associations of maternal vitamin D deficiency with pregnancy and neonatal complications in developing countries: a systematic review. Nutrients..

[4] Edwards MH, Cole ZA, Harvey NC, Cooper C (2014). The global epidemiology of vitamin D status. J Aging Res Clin Prac..

[5] Vandevijvere S, Amsalkhir S, Van Oyen H, Moreno-Reyes R (2012). High prevalence of vitamin D deficiency in pregnant women: a national cross-sectional survey. PloS one..

[6] Puri S, Marwaha RK, Agarwal N, Tandon N, Agarwal R, Grewal K, Reddy DH, Singh S (2008). Vitamin D status of apparently healthy schoolgirls from two different socioeconomic strata in Delhi: relation to nutrition and lifestyle. Br J Nutri.

[7] Marwaha RK, Tandon N, Chopra S, Agarwal N, Garg MK, Sharma B, Kanwar RS, Bhadra K, Singh S, Mani K, Puri S (2011). Vitamin D status in pregnant Indian women across trimesters and different seasons and its correlation with neonatal serum 25-hydroxyvitamin D levels. Br J Nutri.

[8] Divakar H, Singh R, Narayanan P, Divakar GV (2017). Prevalence of Vitamin D Deficiency In A Population Of Indian Women-A Call For Universal Supplementation. J Evid Based Med Healthc.

[9] Jani R, Palekar S, Munipally T, Ghugre P, Udipi S. Widespread 25-hydroxyvitamin D deficiency in affluent and nonaffluent pregnant Indian women. BioMed Res Int. 2014;2014:1-8. Article ID 892162.. 10.1155/2014/892162. 10.1155/2014/892162 PMC4087283 25045711

[10] Finkelstein JL, Duggan C, Mehta S, Thomas T, Srinivasan K, Kurpad AV (2016). Maternal vitamin d status and adverse pregnancy and neonatal outcomes in India. FASEB J.

[11] Mousa A, Abell S, Scragg R, De Courten B. Vitamin D in reproductive health and pregnancy. In Seminars Reprod Med. 2016; 34 (2),. e1-e13). Thieme Medical Publishers. 10.1055/s-0036-1583529 27228115

[12] Wei SQ, Qi HP, Luo ZC, Fraser WD (2013). Maternal vitamin D status and adverse pregnancy outcomes: a systematic review and meta-analysis. J Matern Fetal Neonatal Med.

[13] Liu N, Kaplan AT, Low J, Nguyen L, Liu GY, Equils O, Hewison M (2009). Vitamin D induces innate antibacterial responses in human trophoblasts via an intracrine pathway. Biol Reprod.

[14] Evans KN, Nguyen L, Chan J, Innes BA, Bulmer JN, Kilby MD, Hewison M (2006). Effects of 25-hydroxyvitamin D3 and 1, 25-dihydroxyvitamin D3 on cytokine production by human decidual cells. Biology of reproduction.

[15] Nicolaidou P, Hatzistamatiou Z, Papadopoulou A, Kaleyias J, Floropoulou E, Lagona E, Tsagris V, Costalos C, Antsaklis A (2006). Low vitamin D status in mother-newborn pairs in Greece. Calcified Tissue International.

[16] Dijkstra SH, van BA JJW, de Vleeschouwer LH, Huysman WA, van den Akker EL (2007). High prevalence of vitamin D deficiency in newborn infants of high-risk mothers. Arch Dis Child.

[17] Asaduzzaman M, Basak R, Islam S, Juliana FM, Ferdous T, Islam MJ, Mamun A, Sabrina S, Uddin MM, Mosleh M, Islam K (2018). Vitamin D deficiency and insufficiency in healthy pregnant women living in Dhaka, Bangladesh. IOSR-JDMS.

[18] Zhao Y, Yu Y, Li H, Chang Z, Li Y, Duan Y, Wang J, Jiang S, Yang Z, Yin SA (2017). Vitamin D status and the prevalence of deficiency in lactating women from eight provinces and municipalities in China. PloS one.

[19] Andiran N, Yordam N, Ozon A (2002). Risk factors for vitamin D deficiency in breast-fed newborns and their mothers. Nutrition.

[20] Javaid MK, Crozier SR, Harvey NC, Gale CR, Dennison EM, Boucher BJ, Arden NK, Godfrey KM, Cooper C, Princess Anne Hospital Study Group (2006). Maternal vitamin D status during pregnancy and childhood bone mass at age 9 years: a longitudinal study. The Lancet.

[21] Baggerly CA, Cuomo RE, French CB, Garland CF, Gorham ED, Grant WB, Heaney RP, Holick MF, Hollis BW, McDonnell SL, Pittaway M (2015). Sunlight and vitamin D: necessary for public health. J Am Coll Nutr.

[22] Khan KS, Kunz R, Kleijnen J, Antes G (2003). Five steps to conducting a systematic review. Journal of the Royal Society of Medicine.

[23] Checklist PRISMA statement 2009; Available in http://prisma- statement.org/PRISMA Statement/Checklist.aspx Accessed December 2018

[24] Zhang L, Chow EP, Su S, Yiu WL, Zhang X, Iu KI, Yuan L (2015). A systematic review and meta-analysis of the prevalence, trends, and geographical distribution of HIV among Chinese female sex workers (2000–2011): implications for preventing sexually transmitted HIV. Int J Infect Dis.

[25] Hoy D, Brooks P, Woolf A, Blyth F, March L, Bain C, Buchbinder R (2012). Assessing risk of bias in prevalence studies: modification of an existing tool and evidence of interrater agreement. J Clin Epidemiol.

[26] Assessing risk of bias in included studies - Cochrane Handbook, 2011; Available in http://handbook-51.cochrane.org/chapter_8/8_assessing_risk_of_bias_in_included_studies.htm. Accessed November 2018.

[27] Cochrane Collaboration.RevMan 5.3 user guide. The Cochrane Collaboration. 2014; Available with RevMan, 5.

[28] Sergeant, ESG. Epitools epidemiological calculators 2018. Ausvet Pty Ltd. Available at. http://epitools.ausvet.com.au Accessed November 2018.

[29] Borenstein M, Hedges L, Rothstein H. Meta-analysis: Fixed effect vs. random effects. Meta-analysis. com. 2007.

[30] Ried, K. Interpreting and understanding meta-analysis graphs: a practical guide Reprinted from Australian Family Physician 2006; 35 (8). 16894442

[31] Sachan A, Gupta R, Das V, Agarwal A, Awasthi PK, Bhatia V (2005). High prevalence of vitamin D deficiency among pregnant women and their newborns in northern India. The American journal of clinical nutrition.

[32] Farrant HJ, Krishnaveni GV, Hill JC, Boucher BJ, Fisher DJ, Noonan K (2009). et al. Vitamin D insufficiency is common in Indian mothers but is not associated with gestational diabetes or variation in newborn size. Eur J Clin Nutr.

[33] Sahu M, Bhatia V, Aggarwal A, Rawat V, Saxena P, Pandey A, Das V (2009). Vitamin D deficiency in rural girls and pregnant women despite abundant sunshine in northern India. Clin Endocrinol.

[34] Singla R, Dutta U, Aggarwal N, Bhadada SK, Kochhar R, Dhaliwal LK (2015). Vitamin-D deficiency is associated with gallbladder stasis among pregnant women. Digestive diseases and sciences.

[35] Ajmani SN, Paul M, Chauhan P, Ajmani AK, Yadav N (2016). Prevalence of vitamin D deficiency in burka-clad pregnant women in a 450-Bedded Maternity Hospital of Delhi. J Obstet Gynecol India.

[36] Sharma S, Kumar A, Prasad S, Sharma S (2016). Current Scenario of Vitamin D Status During Pregnancy in North Indian Population. The Journal of Obstetrics and Gynecology of India.

[37] Krishnaveni GV, Veena SR, Winder NR, Hill JC, Noonan K, Boucher BJ, Fall CH (2011). Maternal vitamin D status during pregnancy and body composition and cardiovascular risk markers in Indian children: the Mysore Parthenon Study. Am J Clin Nutr.

[38] Veena SR, Krishnaveni GV, Srinivasan K, Thajna KP, Hegde BG, Gale CR, Fall CH (2017). Association between maternal vitamin D status during pregnancy and offspring cognitive function during childhood and adolescence. Asia Pacific journal of clinical nutrition.

[39] Nandal R, Chhabra R, Sharma D, Lallar M, Rathee U, Maheshwari P (2016). Comparison of cord blood vitamin D levels in newborns of vitamin D supplemented and unsupplemented pregnant women: a prospective, comparative study. J Matern Fetal Neonatal Med.

[40] Kumar P, Shenoi A, Kumar RK, Girish SV, Subbaiah S (2015). Vitamin D Deficiency Among Women in Labor and Cord Blood of Newborns. Indian Pediatr.

[41] Chary AV, Hemalatha R, Seshacharyulu M, Murali MV, Jayaprakash D, Kumar BD (2015). Vitamin D deficiency in pregnant women impairs regulatory T cell function. J Steroid Biochem Mol Biol..

[42] Dasgupta A, Saikia UK, Sarma D (2012). Status of 25 (OH) D levels in pregnancy: A study from the North Eastern part of India. Indian J Endocrinol Metab.

[43] Sablok A, Batra A, Thariani K, Batra A, Bharti R, Aggarwal AR, Chellani H (2015). Supplementation of vitamin D in pregnancy and its correlation with feto-maternal outcome. Clin Endocrinol.

[44] Marwaha RK, Tandon N, Chopra S, Agarwal N, Garg MK, Sharma B, Puri S (2011). Vitamin D status in pregnant Indian women across trimesters and different seasons and its correlation with neonatal serum 25-hydroxyvitamin D levels. Br J Nutr.

[45] Higgins JP, Thompson SG, Deeks JJ, Altman DG. Measuring inconsistency in meta-analyses. Bmj. 2003;327(7414):557–60. 10.1136/bmj.327.7414.557 PMC192859 12958120

[46] Junaid K, Rehman A, Jolliffe DA, Wood K, Martineau AR (2015). High prevalence of vitamin D deficiency among women of child-bearing age in Lahore Pakistan, associating with lack of sun exposure and illiteracy. BMC Women's Health.

[47] Woo J, Lam CW, Leung J, Lau WY, Lau E, Ling X, Rockell JE (2008). Very high rates of vitamin D insufficiency in women of child-bearing age living in Beijing and Hong Kong. Br J Nutr.

[48] Bukhary NBI, Isa ZM, Shamsuddin K, Lin KG, Mahdy ZA, Hassan H, Yeop NSH (2016). Risk factors for antenatal hypovitaminosis D in an urban district in Malaysia. BMC Pregnancy Childbirth.

[49] Tasset JL. A systematic review of vitamin D deficiency in pregnancy in India and its impact on maternal and fetal outcomes.2014;( Doctoral dissertation, University of Cincinnati).

[50] Dovnik A, Mujezinović F, Treiber M, Balon BP, Gorenjak M, Maver U, Takač I (2014). Seasonal variations of vitamin D concentrations in pregnant women and neonates in Slovenia. Eur J Obstet Gynecol Reprod Biol.

[51] Ono Y, Suzuki A, Kotake M, Zhang X, Nishiwaki-Yasuda K, Ishiwata Y, Itoh M (2005). Seasonal changes of serum 25-hydroxyvitamin D and intact parathyroid hormone levels in a normal Japanese population. J Bone Mineral Metab.

[52] Mithal A, Wahl DA, Bonjour JP, Burckhardt P, Dawson-Hughes B, Eisman JA, IOF Committee of Scientific Advisors (CSA) Nutrition Working Group (2009). Global vitamin D status and determinants of hypovitaminosis D. Osteoporos Int.

[53] Contreras-Manzano A, Villalpando S, Robledo-Pérez R (2017). Vitamin D status by sociodemographic factors and body mass index in Mexican women at reproductive age. Salud Pública De México.

[54] Pratumvinit B, Wongkrajang P, Wataganara T, Hanyongyuth S, Nimmannit A, Chatsiricharoenkul S, Manonukul K, Reesukumal K (2015). Maternal vitamin D status and its related factors in pregnant women in Bangkok, Thailand. PloS one.

[55] Azami MILAD, Shamloo MBB, Nasirkandy MP, Veisani YOUSEF, Rahmati SHOBOO, YektaKooshali MH, et al. Prevalence of vitamin D deficiency among pregnant women in Iran: a systematic review and meta-analysis. Koomesh. 2017;19(3):505–14.

[56] Agarwal KS, Mughal MZ, Upadhyay P, Berry JL, Mawer EB, Puliyel JM (2002). The impact of atmospheric pollution on vitamin D status of infants and toddlers in Delhi, India. Arch. Dis. Child..

[57] Van der Meer IM, Karamali NS, Boeke AJ, Lips P, Middelkoop BJ, Verhoeven I, Wuister JD (2006). High prevalence of vitamin D deficiency in pregnant non-Western women in The Hague, Netherlands. Am J Clin Nutr..

[58] Parlak M, Kalay S, Kalay Z, Kirecci A, Guney O, Koklu E (2015). Severe vitamin D deficiency among pregnant women and their newborns in Turkey. The Journal of Maternal-Fetal & Neonatal Medicine..

[59] Roth DE, Shah MR, Black RE, Baqui AH (2010). Vitamin D status of infants in north-eastern rural Bangladesh: preliminary observations and a review of potential determinants. J Health Popul Nutr.

[60] Kruger MC, Kruger IM, Wentzel-Viljoen E, Kruger A (2011). Urbanization of black South African women may increase risk of low bone mass due to low vitamin D status, low calcium intake, and high bone turnover. Nutr Res.

[61] Misra A, Singhal N, Sivakumar B, Bhagat N, Jaiswal A, Khurana L (2011). Nutrition transition in India: Secular trends in dietary intake and their relationship to diet-related non-communicable diseases. Journal of diabetes.

[62] Akhtar S (2016). Vitamin D status in South Asian populations–risks and opportunities. Critical reviews in food science and nutrition.

[63] Anwar S, Iqbal MP, Azam I, Habib A, Bhutta S, Soofi SB, Bhutta ZA (2016). Urban and rural comparison of vitamin D status in Pakistani pregnant women and neonates. J Obstet Gynaecol.

[64] Gupta A (2014). Vitamin D deficiency in India: prevalence, causalities and interventions. Nutrients.

[65] Christesen H, Falkenberg T, Lamont R (2012). The impact of vitamin D on pregnancy: a systematic review. Acta Obstetricia et Gynecologica Scandinavica.

[66] Moghraby SA, Al Shawaf T, Akiel A, Sedrani SH, El Idrissy AT, Al-Meshari AA (1987). Parity and vitamin D metabolites. Ann Trop Paediatr.

[67] Saraf R, Morton S, Camargo CA, Grant CC (2016). Global summary of maternal and newborn vitamin D status–a systematic review. Matern Child Nutr.

[68] Shah I, James R, Barker J, Petroczi A, Naughton DP (2011). Misleading measures in Vitamin D analysis: a novel LC-MS/MS assay to account for epimers and isobars. Nutrition Journal.

[69] Recommended dietary Allowance for Indians. 2010; available in ninindia.org/ dietaryguidelinesforninwebsite.pdf Accessed February 2019.

[70] WHA65, R. 11. Nutrition. Maternal, infant and young child nutrition: draft comprehensive implementation plan. Sixth-fifth World Health Assembly, Geneva, 2012; 21-26. (http://apps.who.int/gb/ebwha/pdf_files/WHA65/A65_11-en.pdf, accessed 18 December 2018).

[71] Holick MF (2007). Vitamin D deficiency. New England Journal of Medicine.

[72] Bandeira F, Griz L, Dreyer P, Eufrazino C, Bandeira C, Freese E (2006). Vitamin D deficiency: a global perspective. Arquivos Brasileiros de Endocrinologia & Metabologia.

[73] Micka AE. Vitamin D status among Bangladeshi women of reproductive age, 2009; (Doctoral dissertation, University of Massachusetts Amherst).

[74] Eyles D, Anderson C, Ko P, Jones A, Thomas A, Burne T, McGrath J (2009). A sensitive LC/MS/MS assay of 25OH vitamin D 3 and 25OH vitamin D 2 in dried blood spots. Clinica Chimica Acta.

